# Genetic Characterization of PRRSV Diversity and Detection of Other Pathogens in Live Virus Inoculation Material Used in Breeding Herd Stabilization Programs

**DOI:** 10.3390/microorganisms14061207

**Published:** 2026-05-27

**Authors:** Mariamawit Z. Mohammed, Daniel C. L. Linhares, Michael A. Zeller, Gustavo S. Silva, Christopher Rademacher, Christina Peterson, Giovani Trevisan

**Affiliations:** Department of Veterinary Diagnostic and Production Animal Medicine, College of Veterinary Medicine, Iowa State University, Ames, IA 50011, USA

**Keywords:** swine, live virus inoculation, porcine reproductive and respiratory syndrome virus, metagenome, next-generation sequencing

## Abstract

Live virus inoculation (LVI) is widely used for porcine reproductive and respiratory syndrome virus (PRRSV) stabilization, yet preparation practices and pathogen composition remain poorly characterized. This study aimed to evaluate variability in LVI preparation, quantify PRRSV genomic load, and detect additional swine pathogens. A survey was conducted to document LVI preparation methods, and samples were analyzed using reverse-transcription quantitative PCR (RT-qPCR) for PRRSV quantification and next-generation sequencing for PRRSV and the metagenomic characterization of additional pathogens. Among 61 LVI samples, substantial variability was observed in preparation practices and viral composition, with 31 distinct PRRSV variants identified and seven samples containing multiple strains. PRRSV RNA concentrations ranged from 10^1.69^ to 2.52 × 10^8^ copies/mL. Metagenomic analysis detected a complete or near-complete genome for PRRSV, porcine parvovirus, and porcine circovirus type 2. Genome fragments of porcine sapovirus, porcine rotavirus, porcine astrovirus, and bacterial genetic material from *Salmonella* spp., *Pseudomonas* spp., *Streptococcus* spp., and *Escherichia coli* were also detected. These findings highlight substantial heterogeneity in LVI materials and encourage the use of next-generation sequencing to verify LVI PRRSV composition and screen for co-existing pathogens, reinforcing the need for standardized preparation protocols and further investigation into optimal viral dosing for effective immunization.

## 1. Introduction

Porcine reproductive and respiratory syndrome virus (PRRSV) is an enveloped, positive-sense, single-stranded RNA virus with a ~15 kb genome that contains at least 10 open reading frames (ORFs) encoding both nonstructural and structural proteins [1]. PRRSV belongs to the family *Arteriviridae*, which includes two species of PRRSV: *Betaarterivirus europensis*, commonly known as PRRSV-1, and *Betaarterivirus americense*, commonly known as PRRSV-2 (International Committee on Taxonomy of Viruses [ICTV], https://ictv.global/report/chapter/arteriviridae/arteriviridae/betaarterivirus, accesses on 23 May 2025). PRRSV causes a devastating reproductive and respiratory disease of swine named after the virus, known as porcine reproductive and respiratory syndrome (PRRS) [1]. In a recent economic analysis of PRRS, the impact on the US swine industry was estimated at $1.2 billion annually [2], $150 million in Canada, and between €75.72 and €650.09 per sow in Europe [3]. The substantial economic burden associated with PRRSV is partly driven by its exceptionally high evolutionary rate (4.71–9.8 × 10^−2^/site/year) [4], which facilitates the emergence of numerous genetic variants, combined with the limited cross-protection conferred by host immunity, making effective disease control challenging [5].

In response to the persistent and evolving challenges posed by PRRSV, the swine industry has adopted a range of control strategies, including modified live virus (MLV) vaccines, enhanced biosecurity protocols, herd closure, and herd stabilization programs [6,7,8]. Among control strategies, MLV vaccination is widely used globally to reduce clinical disease and viral shedding [9], while the usage of live virus inoculation (LVI) has become a commonly used approach in the US [10]. Nowadays, the use of either MLV, LVI, or a combination of both is common in the US swine industry for PRRSV control and elimination programs [11]. MLV and LVI differ in their PRRSV composition characteristics and production nature. MLV vaccines are commercially available vaccines produced under a controlled environment using sterile diluents and consistently containing a single attenuated PRRSV. MLV vaccines are typically used to induce heterologous immunity against circulating wild-type viruses within a herd [12]. In contrast, LVI is produced using field-collected biological samples containing non-attenuated viruses, usually from a recently infected population [13]. LVI is mainly used to develop homologous immunity to the circulating virus, to eliminate the virus from the herd and to produce PRRSV-negative piglets faster [10]. However, the practice is considered illegal in some countries [1].

Despite the frequent usage of LVI [10], in the US swine industry, the composition—including the diversity of PRRSV, the presence of other pathogens, and the variability in preparation practices—has not been systematically investigated. This knowledge gap raises critical concerns about the characteristics of any PRRSV variant(s) present in LVIs, potentially leading to increased clinical implications, viral recombination, exposure to unintended pathogens, and inconsistent outcomes across production systems [12,14,15,16]. This study aimed to document the variation in LVI preparation methods across production systems, comprehensively characterize the genetic diversity of PRRSV present within LVI samples, determine the expected number of PRRSV genomic copies, and investigate the potential presence of additional pathogens of swine health interest in LVI samples used as a response to a PRRSV outbreak in a swine breeding herd.

## 2. Materials and Methods

### 2.1. Overview of the Study Design

This observational study focused on characterizing PRRSV and additional pathogens present in LVI material utilized for PRRSV exposure in US breeding herds from June 2024 to July 2025. Breeding herds in the US using LVI for PRRSV control and exposure programs were recruited to participate regardless of the farm size. A survey was used to capture information on the LVI preparation practices. In efforts to capture information for different practices being used across the swine industry, a production company or veterinary clinic was limited to enroll a maximum of ten LVI samples. Serum samples were collected as part of the routine veterinary practice procedure on the farms from breeding herds that experienced a PRRSV outbreak and subsequently used LVI as a method of herd exposure. One serum sample aliquot was shipped from farms to a veterinary diagnostic laboratory in refrigerated in cooler ice boxes, received at the laboratory and accessioned for diagnostic testing through routine diagnostic processes. Samples were submitted for PRRSV quantification using reverse-transcriptase quantitative polymerase chain reaction (RT-qPCR) and genome sequencing using next-generation sequencing (NGS) with a pathogen discovery approach.

The five US commercially available PRRSV-2 MLV vaccines, namely Prevacent^®TM^ PRRS (Elanco, Indianapolis, IN, USA) (GenBank KU131568.1), PRIME PAC^®^ PRRS RR (Merck Animal Health, Rahway, NJ, USA) (GenBank DQ779791.1), Fostera^®^ PRRS vaccine (Zoetis, Parsippany, NJ, USA) (GenBank AF494042.1), Ingelvac PRRS^®^ MLV (Boehringer Ingelheim, Duluth, GA, USA) (GenBank AF066183.4), and PRRSGard^®^ (Pharmgate, Wilmington, NC, USA), and the formerly commercialized Ingelvac PRRS^®^ ATP (Boehringer Ingelheim, Duluth, GA, USA) (GenBank DQ988080.1) were included as a comparison to the LVI samples. MLVs were reconstituted according to the manufacturer’s instructions and then submitted for RT-qPCR and NGS testing.

### 2.2. LVI Preparation Practices

A survey was used to collect information about LVI preparation practices. The respective herd veterinarians overseeing the preparation of the LVI were asked to complete a survey with information on the sample type used for LVI preparation, route of exposure, dose administered, diluent used, donor animal information, antibiotic usage on LVI preparation, commercial MLV vaccine usage along with LVI, and categories of animals that received the LVI. Dilution procedures captured from this survey were combined with RT-qPCR results and used to estimate the number of viral particles administered and to characterize the diversity of the LVI preparation.

### 2.3. Sample Collection and RT-qPCR Testing

The samples used to prepare the LVI were shipped to the Iowa State University Veterinary Diagnostic Laboratory (ISU-VDL) and tested for PRRSV-1 and PRRSV-2 using commercially available and previously validated RT-qPCR assays [17]. All biological samples were considered positive for PRRSV RNA when the RT-qPCR cycle threshold (Ct) was below 37 [15]. LVI samples positive for PRRSV by RT-qPCR were subsequently submitted for NGS.

### 2.4. Quantification of Estimated Viral Particles Present in the LVI

To estimate the number of genomic copies administered in the LVI, RT-qPCR results were used as input to calculate the estimated PRRSV particles per mL (i.e., RNA copies/mL) following the procedures outlined in the survey completed by the practitioner herd veterinarians. Descriptive statistical analysis was conducted using R version 4.4.1. The term “estimated PRRSV particles per mL” is used hereon, since dilution procedures may yield a final number of viral particles per mL different from the calculated value, and the RT-qPCR assay does not differentiate viable from non-viable PRRSV particles.

### 2.5. Next-Generation Sequencing Procedures

Total nucleic acid extraction and sequence library preparation were conducted as previously described [18]. Briefly, nucleic acids were extracted using the MagMAX^TM^ Pathogen RNA/DNA Kit (Thermo Fisher Scientific, Austin, TX, USA). Double-stranded cDNA was synthesized using the NEXTflex^TM^ Rapid RNA-Seq Kit (Revvity Health Sciences, Waltham, MA, USA). Sequencing libraries were constructed using the Illumina DNA Prep kit (Illumina, San Diego, CA, USA) with dual indexing. Fluorometer Qubit 2.0 (Life Technologies, USA) was used to determine the concentration of the sequencing library, which was then normalized to a concentration of 2 nM. The pooled libraries were sequenced on an Illumina MiSeq i100 platform at the NGS section in the ISU-VDL on a fee-for-service basis using a 600-Cycle Reagent Kit (Illumina, San Diego, CA, USA) to generate 300-base-pair paired-end reads, following standard Illumina protocols.

### 2.6. NGS Assembling Procedures

To assess genetic diversity, NGS-generated raw sequencing reads were processed using Geneious Prime^®^ version 2025.1.2 (https://www.geneious.com, accesses on 23 May 2025). Raw reads were imported and processed using BBDuk Adapter/Quality Trimming (version 38.84) to eliminate low-quality reads and adaptors with a set minimum Phred quality score of 30 and a minimum read length of 100 base pairs [19]. The trimmed reads were evaluated with FastQC and the quality-controlled reads were then de novo assembled using SPAdes assembler [20]. The resulting contigs were first aligned against the *Sus scrofa* genome as a reference (GenBank NC_010443.5) [21] to map and filter out host-derived, i.e., porcine, sequences. Contigs with no significant matches to the *Sus scrofa* genome were subsequently queried against a locally indexed viral genome database (viral.1.1.genomic.fna.gz) downloaded on 1 July 2025 from NCBI GenBank (https://www.ncbi.nlm.nih.gov/) and configured in Geneious Prime (Geneious Prime^®^ version 2025.1.2) for local BLAST analysis. Reads with hits in the viral database were further examined using the BLAST hit table to determine their corresponding viral genus or species identification. Based on these results, a consensus viral genome was reconstructed. A similar pipeline was followed for bacterial identification, except that NCBI BLAST (https://blast.ncbi.nlm.nih.gov/Blast.cgi, accesses on 23 May 2025) was used directly via the web interface rather than via a local database [22]. Only bacterial taxa with high sequencing read counts (≥100 reads) belonging to the same genus were considered to minimize false positive results derived from NGS artifacts and or from other potential reasons like the likelihood of environmental contamination [23]. To assess PRRSV diversity, a PRRSV-specific assembly pipeline was used rather than the metagenomic analysis workflow described above. For PRRSV genome assembly, analyses were conducted in a Linux-based computing environment. Raw sequencing reads were preprocessed using Trimmomatic v0.39 [24] to remove adapter sequences and trim low-quality bases, and reads shorter than 36 nucleotides were discarded. Read quality was assessed using FastQC (v0.12.1), and reads were assembled using SPAdes v3.15.5.

### 2.7. Sequence Alignment and Tree Building

Pathogen-specific whole-genome and PRRSV ORF5 sequences were aligned using the MAFFT algorithm setting’s default strategy of fitting data size (200PAM/k = 2 scoring matrix; gap open penalty: 1.53; offset value: 0.123) [25]. A maximum likelihood phylogenetic tree of assembled PRRSV sequences was generated using RAxML v.8.2.11 with nucleotide model of GTR GAMMA and Rapid hill-climbing algorithm with 1 starting tree and 1 parsimony random seed [26]. The tree was rooted on PRRSV-2 prototype virus [27]. FigTree v1.4.4 (http://tree.bio.ed.ac.uk/software/figtree/, accesses on 23 May 2025) was used for tree visualization. PRRSV sequences were named according to lineages, sublineages [28], and variants [5] based on ORF5 classification terminologies and methodologies. Due to historical usage reference, although no longer used, ORF5-based restriction fragment length polymorphism (RFLP) nomenclature was retained in the ORF5 naming [29].

### 2.8. Recombination Analysis

Recombination analysis was conducted for PRRSV using whole-genome sequences recovered from this study, along with the MLV vaccine viruses and the PRRSV-2 reference virus VR-2332 (GenBank U87392.3) [30]. Genome fragments, i.e., contigs used to detect multiple PRRSVs, were not included in the PRRV recombination analysis. All sequences were compiled into a single database containing a total of 69 PRRSV-2 whole genomes. The aligned dataset was analyzed for recombination events and breakpoint positions using RDP4 software version 4.101 [31]. A recombination event was only considered if it was supported by at least six of the seven default detection methods implemented in RDP4, namely RDP, GENECONV, BootScan, MaxChi, Chimera, SiScan, and 3Seq [15]. Recombinant sequences identified through RDP4 were further confirmed and visualized using SimPlot version 3.5.1 [32].

## 3. Results

A total of 61 LVI samples from 14 production systems across eight US states were obtained (Table 1). In seven farms, exposure to LVI occurred twice, with a re-harvest of serum for the second exposure approximately 4 weeks after the initial exposure (Table 2). For these specific farms, both LVIs (i.e., the first- and second-harvest sera) were included in the study. All 61 LVI materials were prepared from serum samples with no lung sample utilized for any of the samples, of which most were prepared from the pooled serum of multiple piglet donors (72.1%, *n* = 44), while the remaining were derived from single donors (27.9%, *n* = 17). Serum samples originated from suckling piglets (95.1%, *n* = 58) and sows (4.92%, *n* = 3). The number of piglet donors per sample varied widely, ranging from one to 150 donors, with an average of 14 and a median of five. As for the diluent used in LVI preparation, saline was the most common (50.8%, *n* = 31), followed closely by phosphate-buffered saline (PBS) (42.6%, *n* = 26). Sterile water was used in only a few preparations (6.6%, *n* = 4). The majority of LVI preparations (80.3%, *n* = 49) did not include antibiotics. Among those that did include antibiotics, ceftiofur sodium was the most commonly used (83.3%, *n* = 10), followed by enrofloxacin (16.6%, *n* = 2) (Table 1).

Regarding the route of LVI exposure, intramuscular injection was most common (96.7%, *n* = 59), followed by nasal exposure (3.3%, *n* = 2). LVI was administered once (76.21%, *n* = 41), twice (31.15%, *n* = 19), or three times (1.64%, *n* = 1). The second and third LVI doses were given 17 to 36 days after the first LVI dose. In most cases (75.41%, *n* = 46), a combination of replacement gilts, sows, and boars received the LVI. In some cases, only gilts were exposed (11.47%, *n* = 7), while only gilts and sows received the inoculation in others (11.47%, *n* = 7). In a single case (1.64%, *n* = 1), LVI was administered to gilts and boars. LVI was used as the sole immunization strategy in 52.45% of cases (*n* = 32). Some herds received MLV vaccine prior to LVI (27.87%, *n* = 17), and others received both LVI and a killed PRRSV vaccine (16.4%, *n* = 10). LVI was administered at different times following the outbreak, ranging from 2 to 19 weeks.

**Figure 1:**
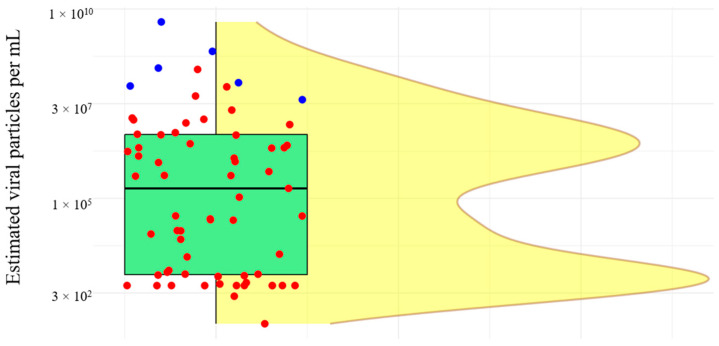
Raincloud plot for the expected number of PRRSV viral particles per mL of LVI (red dots) and MLV vaccines (blue dots). The shaded yellow area represents the distribution of viral particles present in the LVI and MLV samples. The green area represents this LVI distribution according to the quantities using a Box plot format. The *y*-axis is displayed on a log10 scale.

The estimated number of viral particles in LVI samples ranged from 10^1.69^ to 2.52 × 10^8^ PRRSV RNA particles/mL, with a mean of 8.36 × 10^6^ and a median of 3.42 × 10^4^. As a comparison, the genomic quantification of the six PRRSV-2 MLV viruses ranged from 4.03 × 10^7^ to 4.57 × 10^9^ genomic copies/mL (Figure 1).

NGS detected PRRSV RNA in all 61 LVI samples, all of which belonged to the PRRSV-2 species. ORF5 phylogenetic inference for the LVI and MLV (*n* = 6) viruses revealed that the detected viruses were distributed across 12 lineages/sublineages (Table 3).

**Figure 2:**
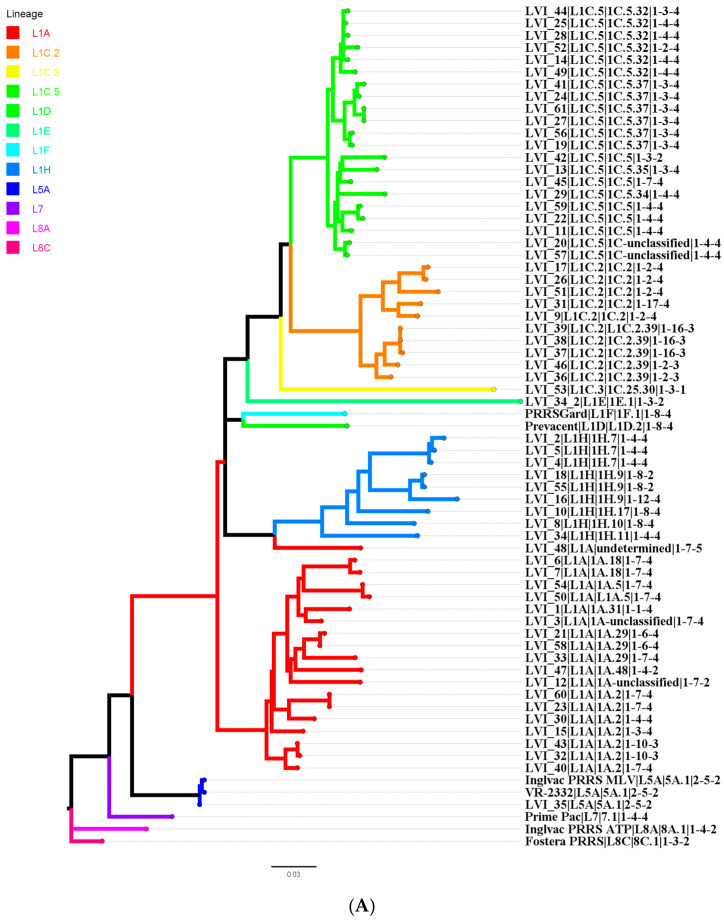

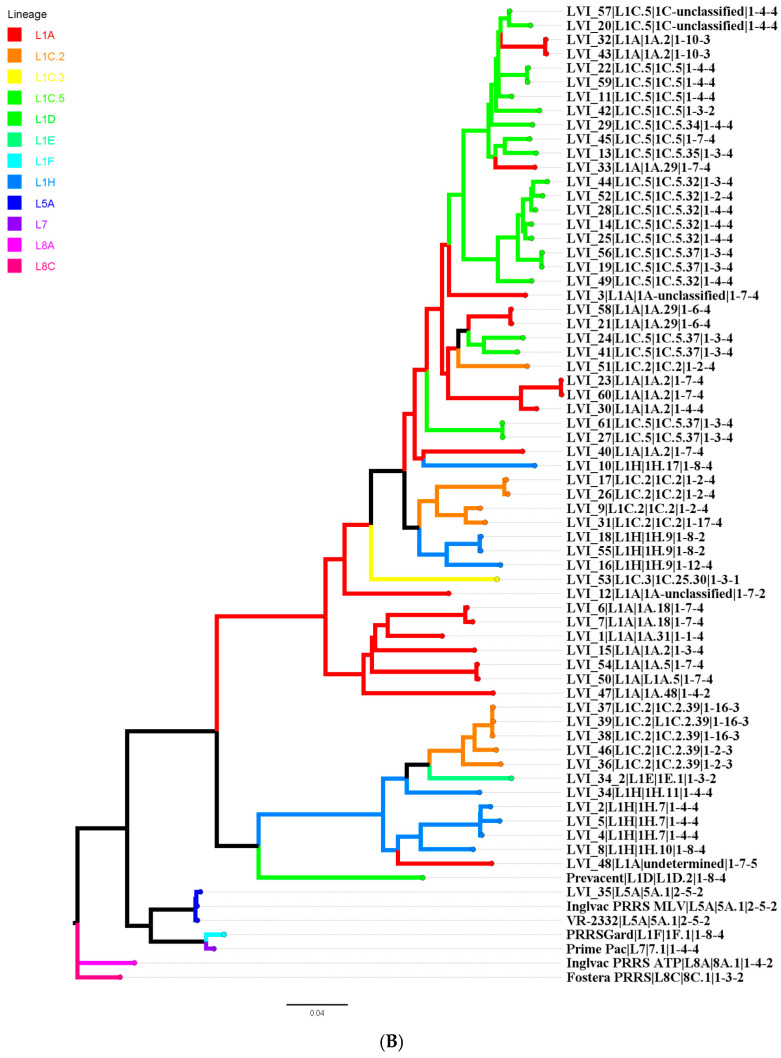
(**A**) Phylogenetic tree of PRRSV sequences of 69 viruses (61 LVI samples, 6 US MLV vaccine-like strains, one additional whole genome recovered from one of the LVI samples, and the PRRSV-2 reference strain VR-2332) based on the ORF5 gene. The tree branch is colored by lineage. Tip labels are formatted as LVI_identifier|lineage/sublineage|variant|RFLP corresponding to each virus. MLVs belong to lineages L1D, L1F, L5A, L7, L8A, and L8C. (**B**) Phylogenetic tree of 69 PRRSV whole-genome sequences. The tree branch is colored by lineage based on ORF5 lineage classification. Lineage, sublineage, and RFLP were assigned based on the ORF5 gene without using the whole-PRRSV genome.

One LVI sample (LVI_34) contained two distinct wild-type PRRSV-2 whole genomes of L1H and L1E lineages. In total, the PRRSV viruses were grouped into 31 distinct variants based on ORF5 classification. The phylogenetic trees constructed from the ORF5 gene (Figure 2A) and the whole-PRRSV genome (Figure 2B) showed incongruent topologies, indicating that the evolutionary relationships inferred from ORF5 alone (Figure 2A) do not fully reflect those observed at the whole-genome level (Figure 2B).

**Figure 3:**
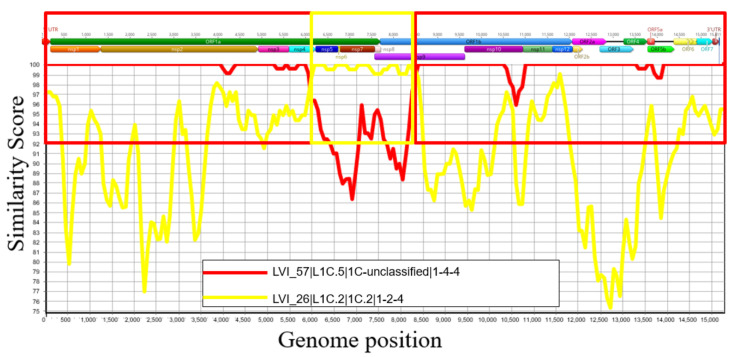
Recombination detected within an LVI recovered virus, used as a query sequence against two wild-type viruses from lineage L1C.5 and L1C.2 PRRSV strains. PRRSV genome regions are presented at the top of the plot. Red and yellow boxes outline the recombination breakpoint events and genome regions derived from each parental virus. Genome regions were plotted in Geneious, and recombination events were determined using SimPlot.

A single PRRSV strain was identified in 91.8% (54 of 61) of the LVI samples. Of these, 98.14% (53 of 54) contained wild-type viruses, while 1.85% (1 of 54) contained an MLV-like strain. The remaining 11.47% (7 of 61) of samples included multiple PRRSV strains. Among these, 85.71% (6 of 7) samples had two distinct strains, whereas 71.42% (5 of 7) contained two wild-type viruses, and 14.28% (1 of 7) contained a combination of a wild-type and an MLV-like virus. In 14.28% (1 of 7) of the LVI samples with more than one PRRSV, three genetically distinct wild-type strains were detected. The recombination analysis performed on assembled PRRSV whole genomes revealed that one of the viruses used in an LVI (LVI_20) is a recombinant virus with parental lineages/sublineages identified as an L1C.5 (LVI_57) and an L1C.2 (LVI_26) recovered from other farms from the same production system (Figure 3).

NGS analysis detected genomic material from a variety of non-PRRSV pathogens. Despite the detection of various genomic fragments for different agents, only those with swine health importance are reported. Bacterial sequences from four genera were recovered. Multiple gene fragments of *Salmonella* spp. (19.67%, *n* = 12), *Pseudomonas* spp. (3.27%, *n* = 2), *Streptococcus* spp. (1.63%, *n* = 1), and *Escherichia* coli (*E. coli*) (1.63%, *n* = 1) were detected. Among the samples, *Salmonella* spp. (3.33 × 10^3^–9.85 × 10^4^ reads), *Pseudomonas* spp. (5.66 × 10^4^–5.79 × 10^4^ reads), *Streptococcus suis* (1.22 × 10^4^ reads), and *E. coli* (3.39 × 10^4^ reads) were identified.

**Figure 4:**
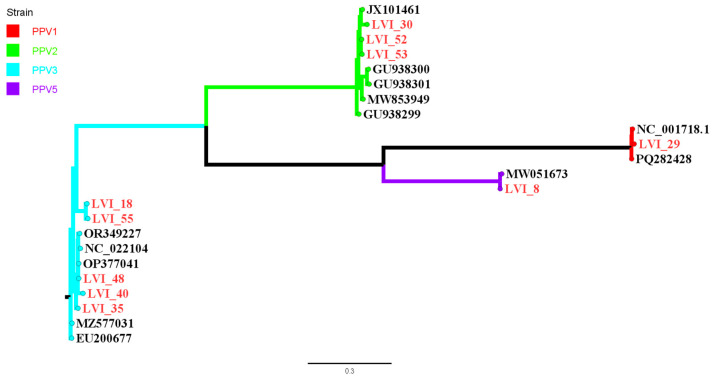
Phylogenetic relationships of porcine parvovirus (PPV) strains identified in this study, along with representative sequences retrieved from GenBank. Sequences recovered in this study are indicated in red. Sequences identified in black were downloaded from GenBank, and the tip label represents the GenBank accession ID.

In addition to PRRSV, other viral sequences were also identified, representing four RNA virus families (*Sedoreoviridae*, *Orthomyxoviridae*, *Caliciviridae* and *Astroviridae*) and two DNA virus families (*Parvoviridae* and *Circoviridae*). Ten samples contained parvovirus (Table 4) belonging to four parvovirus types, PPV1, PPV2, PPV3, and PPV5 (Figure 4). Porcine astrovirus 4 (PAstV-4) was recovered from one sample, with approximately 97.1% of a 6Kb long genome coverage with 93.6% identity to a reference virus (GenBank KU764486) isolated from Oklahoma [33]. Additionally, another member of the *Astroviridae* family porcine astrovirus 5 (PAstV-5) was detected in a separate sample. A 1768 bp long porcine circovirus type 2 (PCV2) genome was recovered and annotated with 97.5% nucleotide identity to a PCV2a virus first identified in the United States in the late 1990s (AF055391.1) [34].

Segments from two species within the rotavirus genus were recovered, including *Rotavirus alphagastroenteritidis*, formerly known as rotavirus A, and *Rotavirus tritogastroenteritidis*, formerly known as rotavirus C. Fragments corresponding to the VP6 gene of *Rotavirus tritogastroenteritidis* with 65.7% coverage were mapped to a reference segment gene GenBank MT771564. Similarly, VP6 gene coverage of 50.5% for *Rotavirus alphagastroenteritidis* were mapped to a reference gene GenBank KY053212 [35]. Further classification was not performed due to the incomplete nature of the recovered segment. In another sample, Porcine sapovirus (SaV) with 78.3% coverage to a 7.3Kb long reference genome (GenBank MW316747) was recovered [36].

Multiple genomic fragments belonging to the genus *orthoreovirus* were detected in three samples. However, due to the incomplete nature of the recovered sequences, it was not possible to assign them to a specific species within the genus. Fragments corresponding to the Influenza virus were identified in three samples. As with the *orthoreoviruses*, the incomplete viral genome fragments prevented classification at the species or subtype level.

## 4. Discussion

This study highlights the considerable variation in how live PRRSV inoculation is currently being prepared and administered across swine production systems in the US. In this study, a total of 61 LVI samples from 14 production systems were collected and tested using RT-qPCR and NGS. Each sample was prepared using different procedures, leading to variability in important factors, such as the estimated number of genomic copies delivered per dose. Since the study was conducted as an exploratory discovery approach on samples used to prepare the LVI material, no final confirmation of the viability of different viruses or bacteria encountered by NGS was pursued.

Previous experimental work has shown that a dose of approximately 10^4.0^ TCID_50_/mL of PRRSV (VR-2332) can cause infection via the intranasal route. In the same study, a dose of 10^2.2^ TCID_50_/mL administered intramuscularly was sufficient to infect all inoculated animals [37]. Another study reported that less than 10 FFU_50_/mL of the ISU-P isolate (GenBank EF532816.1) was enough to induce infection through intramuscular exposure, and that 10^2^ virions were sufficient to cause infection following intranasal administration [38]. A study comparing three different lineages/sublineages of PRRSV-2 (i.e., L1A, L1C.5, and L9A) found that the minimum infectious dose was 10^1.5^–10^1.8^ TCID_50_ [39]. In our study, 31 PRRSV variants (12 lineages/sublineages) were administered at doses ranging from 10^1.69^ genomic copies to over 2.52 × 10^8^ genomic copies, as determined by RT-qPCR. While RT-qPCR provides an estimate of viral genome quantity, there is no direct conversion to TCID_50_, which measures viable infectious particles. However, studies have suggested that the infectious dose can be several logs lower than the genomic copy number, depending on factors such as the virus lineage and the stage of the infection [1,40].

The detection of multiple PRRSV variants within individual LVI samples in this study provides evidence that PRRSV co-infection occurs in the field and, as a consequence, may contribute to PRRSV viral recombination. For a PRRSV recombination event to occur, there is a need for two PRRSVs to infect and replicate in the same cell simultaneously [41]. The administration of multiple viruses through LVI can create the possibility for such simultaneous exposure. A limitation of this study was the lack of prospective follow-up of the enrolled herds, which prevented the identification of recombinant emergent viruses within each farm. Here, the findings indicated that 11.47% of LVI materials contained more than one PRRSV variant, and one sample harbored a recombinant virus. The current study’s findings are consistent with another study that found 90% of the breeding farms studied had at least two PRRSVs, and 59% (12 out of 20) farms had an outbreak with a recombinant PRRSV derived from two wild-type PRRSVs or a wild-type PRRSV and an MLV-like virus [14]. Importantly, their study also demonstrated that herds with multiple PRRSVs and recombinant viruses experienced significantly greater losses (1837 and 1827 more piglet losses per 1000 sows, respectively) compared to herds infected with a single PRRSV and no recombination event.

The findings encountered in this study reinforce concerns that introducing two or more genetically diverse viruses through LVI may lead to unanticipated consequences, including worsened production metrics, unwanted flare-ups, and prolonged outbreaks; however, our study did not include measurements of such outcomes, and therefore, additional research is warranted to understand the consequences of LVI containing more than one distinct PRRSV. Recombination not only complicates diagnostic interpretation but also increases the risk of generating novel variants with altered pathogenicity or immune-escape potential, as demonstrated by the recent L1C.5 PRRSV-2 in the US [42] or the PRRSV-1 Rosalia in Europe [43,44]. LVI is prepared from samples collected from the population that will receive it and is very likely to contain pathogens circulating in that population. The concern with administering LVI with multiple viruses is that it exposes a much larger proportion of the population, many of whom may not be infected with any virus at the time of LVI administration, to multiple viral variants simultaneously. This may increase the likelihood of recombination and, therefore, raise the probability of generating a more fit virus. The detection of recombination using NGS alone is insufficient to definitively confirm its presence; virus isolation and plaque purification would be required for confirmation. However, these procedures were beyond the scope and budget of the present study.

This study also detected the presence of five additional viral families with known relevance to swine health in the LVI samples. These include *Sedoreoviridae*, *Orthomyxoviridae*, *Caliciviridae*, *Astroviridae*, *Parvoviridae*, and *Circoviridae*. Porcine parvovirus is a significant cause of reproductive failure in swine and is most commonly associated with stillbirths, mummification, embryonic death, and infertility (SMEDI) syndrome [1]. Currently, there are eight known PPVs (PPV1-PPV8) in swine [1]. In this study, complete PPV types PPV1, PPV2, PPV3, and PPV5 were identified. The identification of PPV in 10 LVI samples underscores its potential importance and the need for further investigation as a common contaminant of LVI material.

Influenza A virus belongs to the family *Orthomyxoviridae*, genus *Alphainfluenzavirus*, and is a segmented, negative-sense, single-stranded RNA virus capable of infecting a wide range of avian and mammalian species and is commonly detected in swine [1]. The IAVs are not commonly found in the bloodstream. The identification of IAV genetic material fragments in LVI samples is an unexpected finding that deserves further investigation to understand what is occurring during sampling events. One could hypothesize that this observation may be explained by PRRSV-induced immunosuppression, which could facilitate the translocation of IAV from the respiratory tract into the bloodstream. Alternatively, and more pertinent, the detection of IAV in blood could be due to tracheal puncture or an unreported exsanguination technique during the LVI sample collection. The same concern could also apply to other pathogens whose genetic material was detected in LVI but was unexpected or present in very low amounts.

*Salmonella* spp. are rod-shaped, Gram-negative bacteria that cause *salmonellosis*, a major enteric disease affecting swine and capable of causing bacteremia. In addition to veterinary significance, *Salmonella enterica* is recognized as an important foodborne pathogen [45]. *Pseudomonas* spp. are Gram-negative opportunistic pathogens of humans, animals, and plants [46]. Although the detection of these potentially harmful pathogens in the LVI samples indicates the extent and diversity of additional microorganisms present, the identification and reporting of these pathogens in this study relied exclusively on NGS data. Therefore, these findings do not confirm the presence of viable organisms capable of replication or transmission.

The presence of these pathogens alongside PRRSV in the LVI may exacerbate clinical signs and further compromise the herd’s immune system, potentially leading to greater losses. In this study, one farm with PPV in the LVI provided production data, including the weekly number of mummies, and showed that it took 16 weeks to return to baseline levels for mummies and stillbirths, as before the PRRSV outbreak and exposure with LVI. Empirical observations have shown that mummies return to baseline levels earlier after a PRRSV outbreak. This finding warrants further exploration of the dynamics and clinical implications of the two viruses when unintentionally disseminated to the whole herd through LVI. However, production data collection was not part of this study, and group comparisons could not be made.

The identification of non-LVI-target pathogens emphasizes the need for heightened scrutiny when preparing and deploying LVI materials. In a study comparing the resistance of different swine viral pathogens to Ultraviolet-C radiation for inactivation, it was shown that non-enveloped viruses have higher 4D values (the dose needed to inactivate 4 logs of viral load) than enveloped viruses, such as PRRSV [47]. These findings provide actionable insights for veterinarians and swine health professionals, reinforcing the importance of quality control in LVI-based stabilization protocols.

This study underlines the complexity and variability of LVI materials currently used in swine production and highlights the potential risks associated with administering genetically diverse or poorly characterized viral preparations. The detection of multiple PRRSV variants, including recombinant viruses, and the presence of other swine pathogens not intentionally targeted by LVI raise important concerns for both animal health and production outcomes. These findings support the need for standardized protocols in LVI preparation and the integration of molecular surveillance tools such as whole-genome sequencing. Moving forward, efforts to optimize LVI practices should prioritize both efficacy and biosafety, ensuring that tools intended for stabilization do not inadvertently compromise herd health.

Potential approaches that practitioners could use include testing LVI samples using screening techniques such as PCR or culture before using the LVI material to rule out the presence of other pathogens of interest. Genetic sequencing could be used to rule out the presence of MLV-like viruses in LVI samples. If a virus recovered from an LVI preparation is MLV-like, it may be more appropriate to use MLV instead of LVI. Taken together, these findings underscore the urgent need to screen LVI samples for pathogens of interest, such as PPV and PCV2, and, whenever possible, to implement whole-genome sequencing and lineage characterization as part of routine LVI safety screening to minimize the risk of compounding disease burdens through unintended viral diversity.

## Figures and Tables

**Table 1:** Summary of the survey results for LVI preparation characteristics for 61 LVI samples, where swine health professionals were asked to characterize the practices implemented in LVI preparation.

Donor Animal Type	Number	Percentage
Piglet donor	58	95.1
Sow donor	3	4.92
Pooled sample		
LVI pooled from multiple donor animals	44	72.1
LVI from a single donor animal	17	27.9
Diluent used		
Saline	31	50.8
Phosphate-buffered saline (PBS)	26	42.6
Sterile water	4	6.6
Antibiotic added to the LVI		
No antibiotic used	49	80.33
Ceftiofur sodium	10	16.39
Enrofloxacin	2	3.28
**Route of LVI exposure**		
Intramuscular	59	96.7
Nasal	2	3.3
Group of animals that received the LVI		
Replacement gilts, sows, and boars	46	75.41
LVI along with a killed vaccine	9	19.6
MLV vaccine followed by LVI	8	17.4
MLV vaccine followed by LVI and killed vaccine	1	2.2
Two MLV vaccines followed by LVI and another MLV vaccine	1	2.2
LVI only	27	58.7
Only gilts	7	11.47
MLV vaccine followed by LVI	6	58.7
LVI only	1	14.3
Gilts and sows	7	11.47
MLV vaccine followed by LVI	3	42.9
LVI along with a killed vaccine	1	14.3
LVI only	3	42.9
Gilts and boars	1	1.64
LVI only	1	100
Program of vaccine exposure along with LVI		
One LVI dose	22	40.74
MLV vaccine followed by one LVI dose	16	29.62
Two LVI doses	6	11.11
Two LVI doses with killed vaccine	5	9.25
One LVI dose with killed vaccine	1	1.85
MLV vaccine followed by one LVI dose and killed vaccine	1	1.85
Two MLV vaccines followed by one LVI dose and another MLV vaccine	1	1.85
MLV vaccine followed by two LVI doses	1	1.85
Three LVI doses with killed vaccine	1	1.85

**Table 2:** PRRSV genetic characteristics detected in the first and second re-harvest of samples for LVI preparation for a second exposure occurring approximately four weeks after the initial exposure across seven farms, along with additional genetic material indicating bacterial and other viral pathogens identified in both rounds.

PRRSV ORF5-Based Lineages Recovered in the First and Second LVI Harvesting, the Level of Nucleotide Identity Between Them, and the Presence of Additional PRRSV	Viral or Bacterial Pathogen Detected in 1st LVI Sample	Viral or Bacterial Pathogen Detected in the 2nd LVI Sample
L1H, 99.81, and contigs of a second PRRSV in the second harvest	Porcine parvovirus 3, Rotavirus C	Porcine parvovirus 3, *Salmonella* spp.
L1C.5, 99.92, and contigs of a second PRRSV in the second harvest	Porcine Sapovirus, *Salmonella* spp.	*Salmonella* spp.
L1C.5, 98.56, and contigs of a second and third PRRSV in the second harvest	none	Influenza A virus
L1A, 99.98	*Salmonella* spp.	*Salmonella* spp.
L1C.5, 99.89	*Salmonella* spp.	Avian orthoreovirus, *Salmonella* spp.
L1A, 99.94	*Salmonella* spp.	Avian orthoreovirus, *Salmonella* spp.
L1C.5, 99.97	Influenza A Virus, *Salmonella* spp.	none

**Table 3:** Distribution of porcine and reproductive respiratory syndrome virus (PRRSV) classified by lineages identified in live virus inoculum (LVI) material and vaccine samples, including the number and percentage for each genetic classification.

PRRSV Characteristics	Lineage/Sublineage	Number	Percentage
Wild-type	L1C.5	21	29.41
	L1A	19	28.35
	L1C.2	10	14.92
	L1H	9	13.43
	L1C.3	1	1.49
	L1E	1	1.49
Modified live vaccine	L5A	2 (1 from LVI, 1 MLV)	2.98
	L1D	1	1.49
	L1F	1	1.49
	L7	1	1.49
	L8A	1	1.49
	L8C	1	1.49

**Table 4:** Summary of complete parvovirus genomes recovered from LVI samples.

Strain	LVI_ID	No. of Total Raw Reads	No. of Reads Mapped	Total Base Assembled
PPV1	LVI_29	1,102,840	1049	5297
PPV2	LVI_30	867,932	174	5560
PPV2	LVI_52	1,022,112	1420	5509
PPV2	LVI_53	3,574,930	421	5576
PPV3	LVI_18	1,014,108	416	5421
PPV3	LVI_55	890,860	212,152	6937
PPV3	LVI_35	1,203,134	832	5543
PPV3	LVI_40	1,352,462	263,333	6711
PPV3	LVI_48	625,646	5773	5430
PPV5	LVI_8	1,589,274	8337	5936

## Data Availability

The data presented in this study are available on request from the corresponding author, as farm-specific data is business proprietary information and is not available..

## Abbreviations

The following abbreviations are used in this manuscript:

FFU	Fluorescent Focal Units
IACUC	Animal Care and Use Committee
ICTV	International Committee on Taxonomy of Viruses
LVI	Live Virus Inoculation
MLV	Modified Live Virus
NGS	Next-Generation Sequencing
PPRSV	Porcine Reproductive and Respiratory Syndrome Virus
RT-qPCR	Reverse-Transcriptase Quantitative Polymerase Chain Reaction
